# Neonates at Risk: Understanding the Impact of High-Risk Pregnancies on Neonatal Health

**DOI:** 10.3390/medicina61061077

**Published:** 2025-06-11

**Authors:** Rozeta Sokou, Alexandra Lianou, Maria Lampridou, Polytimi Panagiotounakou, Georgios Kafalidis, Styliani Paliatsiou, Paraskevi Volaki, Andreas G. Tsantes, Theodora Boutsikou, Zoi Iliodromiti, Nicoletta Iacovidou

**Affiliations:** 1 Neonatal Department, National and Kapodistrian University of Athens, Aretaieio Hospital, 11528 Athens, Greece; gkafalidis@gmail.com (G.K.); stpaliatsiou@yahoo.gr (S.P.); v.volaki@hotmail.com (P.V.); theobtsk@gmail.com (T.B.); ziliodromiti@yahoo.gr (Z.I.); niciac58@gmail.com (N.I.); 2 Neonatal Intensive Care Unit, “Agios Panteleimon” General Hospital of Nikea, 18454 Piraeus, Greece; alexlianou95@gmail.com (A.L.); marialampridou1@hotmail.com (M.L.); ppppolytimi04@gmail.com (P.P.); 3 Microbiology Department, “Saint Savvas” Oncology Hospital, 11522 Athens, Greece; andreas.tsantes@yahoo.com; 4 Laboratory of Haematology and Blood Bank Unit, School of Medicine, “Attiko” Hospital, National and Kapodistrian University of Athens, 12462 Athens, Greece

**Keywords:** high-risk pregnancies, neonates, preeclampsia, gestational diabetes mellitus, congenital infections, substance use, SARS-CoV-2 infection, fetal growth restriction, prematurity

## Abstract

High-risk pregnancies (HRPs) constitute a significant global health issue due to their strong association with increased maternal and neonatal morbidity and mortality. Although pregnancy is generally characterized by positive expectations, the presence of maternal comorbidities, gestational complications, or adverse socioeconomic and environmental conditions can markedly elevate the probability of unfavorable outcomes. HRPs contribute disproportionately to complications such as preterm birth, fetal growth restriction, low birth weight, and congenital anomalies, which are key determinants of neonatal mortality and long-term developmental and health challenges. A broad spectrum of risk factors as well as insufficient prenatal care, underscores the complex nature of HRPs. These conditions necessitate a multidisciplinary management approach encompassing early risk identification, continuous monitoring, and individualized interventions. The neonatal prognosis in such contexts is strongly influenced by gestational age at delivery, birth weight, the standard of neonatal care, and the underlying etiological factors driving preterm or complicated deliveries. Preventive strategies including comprehensive prenatal screening, systematic antenatal follow-up, and timely referral to specialized perinatal care centers are essential for reducing the burden of HRPs. Furthermore, addressing social determinants of health—such as low socioeconomic status and limited access to healthcare—is critical for optimizing maternal and neonatal outcomes. This review consolidates current evidence on the epidemiology, etiological factors, and clinical implications of high-risk pregnancies, emphasizing the necessity of an integrative, preventive, and multidisciplinary framework to mitigate adverse neonatal outcomes and improve long-term health trajectories.

## 1. Introduction

Pregnancy is a period of profound joy and anticipation, for women and their families. However, it also has significant implications for physical, mental, emotional, and socioeconomic health of the pregnant woman. While most pregnancies result in favorable outcomes, certain cases may be accompanied by considerable uncertainty as to the outcome, necessitating specialized care for both the mother and the fetus. Pregnancies classified as high-risk pregnancies (HRPs) are associated with an increased likelihood of complications and account for approximately 70–80% of pregnancy-related morbidity and mortality [1]. A HRP is defined as a pregnancy in which the presence of one or more identified risk factors increases, in comparison to the general or reference population, the probability of developing severe complications for both the mother and the fetus [1]. These risk factors may stem from pre-existing maternal conditions, complications arising during gestation, or socioeconomic and environmental determinants.

According to the international literature, approximately 10–20% of pregnancies are classified as high-risk [1,2]. HRPs represent a significant public health challenge, as they require specialized healthcare services. The prevention and effective management of such cases comply to the Sustainable Development Goals (SDG 3) of the World Health Organization (WHO), which aim at improving maternal and child health [3]. Globally, over 20 million women face the challenges of a high-risk pregnancy each year. Approximately 830 pregnancy-related deaths occur daily, 99% of which occur in developing countries [4,5]. The incidence is notably higher among pregnant women living in rural areas and among adolescent mothers, emphasizing the critical need for enhanced care and support for vulnerable populations [5].

A substantial proportion of high-risk pregnancies are associated with increased rates of preterm birth, low birth weight, intrauterine growth restriction, and congenital anomalies. These conditions significantly contribute to elevated perinatal mortality and are linked to neurodevelopmental disorders and increased risk of chronic diseases in adult life [1,2]. Thus, early identification of women at increased risk for pregnancy-related complications is of critical importance.

This review consolidates current evidence on the epidemiology, risk factors, and clinical challenges of high-risk pregnancies, with a focus on their impact on both short- and long-term outcomes in offspring. It emphasizes the importance of a comprehensive, multidisciplinary approach to maternal and fetal care to improve long-term health outcomes.

### Methodology and Eligibility Criteria

A systematic literature search was conducted across peer-reviewed databases, including PubMed, Medline, and Scopus, to identify relevant studies addressing high-risk pregnancies, maternal risk factors, and their impact on offspring outcomes. The search strategy utilized a combination of Medical Subject Headings (MeSH) and relevant text keywords to comprehensively capture studies related to pregnancy complications and long-term health consequences in offspring.

Eligible studies comprised clinical trials, cohort studies, case-control studies, and other observational research published in English. Inclusion criteria required that studies report maternal risk factors detectable during routine clinical care and provide data on short- and/or long-term offspring outcomes, including mortality, morbidity, respiratory distress syndrome (RDS), intraventricular hemorrhage (IVH), neurodevelopmental disorders, and gastrointestinal morbidities.

Exclusion criteria included case reports, case series, animal studies, and review articles lacking original data. Study selection was carried out in three stages: initial title screening, abstract review, and full-text evaluation to assess relevance and methodological quality. Due to the narrative design of this review, data were synthesized qualitatively to underscore clinical challenges, identify gaps in current knowledge, and propose avenues for future research.

## 2. Risk Factors Contributing to High-Risk Pregnancies

**Figure 1:**
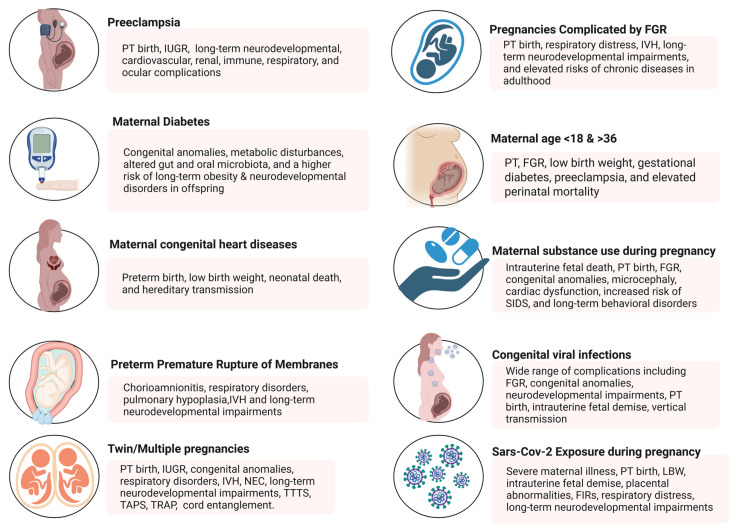
Visual summary of the risk factors that contribute to high risk pregnancies. PT: preterm, IUGR: intrauterine growth restriction, IVH: intraventricular hemorrhage, NEC: necrotizing enterocolitis, TTTS: Twin-to-Twin Transfusion Syndrome, TAPS—Twin Anemia-Polycythemia Sequence, TRAP—Twin Reversed Arterial Perfusion sequence. Created in BioRender. L., A. https://BioRender.com/p2iztwi (accessed on 14 May 2025).

High-risk pregnancies arise from a complex interplay of maternal, fetal, and environmental factors that significantly increase the likelihood of adverse outcomes for both mother and fetus (Figure 1).

### 2.1. Hypertensive Disorders During Pregnancy

Hypertensive disorders during pregnancy—such as chronic hypertension, gestational hypertension, preeclampsia/eclampsia, and preeclampsia superimposed on chronic hypertension—complicate up to 10% of pregnancies worldwide [6]. Preeclampsia, the most severe clinical manifestation of these disorders, affects approximately 3–8% of pregnancies and is a leading cause of maternal and perinatal morbidity and mortality, contributing to 8–10% of all preterm births [7,8,9,10].

As per the current International and American College of Obstetricians and Gynecologists’ Committee guidelines, hypertension in pregnancy is defined as a systolic blood pressure ≥ 140 mmHg or a diastolic blood pressure ≥ 90 mmHg [11,12]. Chronic hypertension refers to elevated blood pressure present before conception or prior to 20 weeks of gestation. Gestational hypertension denotes new-onset hypertension after 20 weeks without accompanying proteinuria or systemic signs of end-organ involvement. Preeclampsia is characterized by hypertension arising after 20 weeks with proteinuria and/or evidence of renal, hepatic, neurological, or hematological dysfunction, or fetal growth restriction. When these preeclamptic features occur in a woman with preexisting chronic hypertension, the condition is classified as preeclampsia superimposed on chronic hypertension [11,12]. The gestational hypertension encompasses a wide range of cases, including those in which women later meet the diagnostic criteria for preeclampsia, as well as individuals with pre-existing elevated blood pressure who are initially misclassified due to the natural decline in blood pressure during the early stages of pregnancy. In most cases, however, proteinuria does not develop, the condition follows a relatively uncomplicated course, and blood pressure typically returns to normal levels postpartum. In such cases, the diagnosis is revised from gestational hypertension to transient hypertension of pregnancy [9,10].

Preeclampsia is a multisystemic, progressive disorder that typically manifests after 20 weeks of gestation or during the postpartum period. It is defined as the onset of de novo hypertension accompanied by signs of maternal organ dysfunction. These include proteinuria > 300 mg/24 h, hematologic complications (e.g., thrombocytopenia with platelet count <150,000/μL, hemolysis), alterations in biochemical biomarkers, abnormal activation of coagulation (disseminated intravascular coagulation), liver involvement (elevated liver transaminases, typically >40 IU/L, with or without upper abdominal pain), neurological complications (such as seizures, altered consciousness, visual disturbances, or stroke) and indications of uteroplacental dysfunction, as evidenced by intrauterine growth restriction (IUGR), abnormal umbilical artery Doppler findings or stillbirth [9,13]. Preeclampsia is one of the most serious pregnancy complications, with significant impact on maternal and perinatal health. It occurs in approximately 3–8% of pregnancies worldwide and is estimated to contribute to over 50,000 maternal deaths and 500,000 neonatal deaths annually [9,12]. Furthermore, preeclampsia is associated with 25% of cases of intrauterine growth restriction, 33% of preterm births, and 20% of admissions to Neonatal Intensive Care Units (NICUs). It represents a major contributor to morbidity and mortality, underscoring the critical importance of early diagnosis and effective clinical management [9]. HELLP syndrome occurs in approximately 10–20% of pregnancies complicated by severe preeclampsia or eclampsia and is characterized by the triad of hemolysis, elevated liver enzyme levels, and thrombocytopenia (low platelet count) [9].

On a global scale, hypertensive disorders of pregnancy complicate approximately 3–10% of all pregnancies and represent a leading cause of maternal and perinatal complications [7]. HDP are responsible for an estimated 18% of maternal deaths worldwide, accounting for approximately 62,000–77,000 deaths annually. Multiple studies conducted in developed and developing countries have demonstrated that HDP are associated with increased rates of adverse perinatal outcomes, including fetal and neonatal death, preterm birth, low birth weight, and perinatal asphyxia [14,15]. Emerging evidence suggests that HDP and particularly preeclampsia, are linked to unfavorable long-term outcomes, and to an elevated risk of neurodevelopmental disorders in the offspring. These conditions have been recently identified as significant perinatal risk factors for lasting neurodevelopmental impairments, independent of gestational age or birth weight In a meta-analysis conducted by Gillian M. Maher to investigate the associations between maternal hypertensive disorders of pregnancy and risks in offspring of autism spectrum disorders (ASDs), attention-deficit/hyperactivity disorder (ADHD), intellectual disability (ID), as well as variations in overall cognitive performance, 61 studies were ultimately included. Among these, twenty studies examined ASD, with 11 reporting adjusted estimates showing a pooled odds ratio (OR) of 1.35 (95% CI: 1.11–1.64), indicating a modestly increased risk. Ten studies assessed ADHD, with six providing adjusted estimates and a pooled OR of 1.29 (95% CI: 1.22–1.36). No significant differences were observed between types of hypertensive pregnancy disorders (e.g., preeclampsia versus other hypertensive disorders) for either ASD or ADHD. For other neurodevelopmental disorders, the results were inconsistent, showing no clear patterns of association [14].

The intrauterine environment in preeclampsia appears to influence the structure and function of the central nervous system, potentially disrupting fetal brain development [16]. Although the precise mechanisms underlying the association between preeclampsia and neurodevelopmental outcomes remain under investigation [16,17], inflammation and oxidative stress—key pathophysiological features of preeclampsia—are believed to play a critical role. These processes affect maternal, placental, and fetal circulation, exposing the fetus to maternal immune system activation and elevated levels of pro-inflammatory cytokines. Consequently, the fetal brain is subjected to harmful stimuli that may interfere with neuronal development [17]. The combined effects of placental hypoxia and ischemia, oxidative stress, inflammation, angiogenic imbalance and altered growth factor expression, may ultimately contribute to disruptions in neuroanatomy and cerebrovascular development [18,19,20]. These alterations have been associated with an increased risk of neurodevelopmental disorders such as ASDs, ADHD, ID, and variations in overall cognitive performance in offspring [20].

Furthermore, preeclampsia has been linked to an increased risk of psychiatric conditions in the offspring, including emotional and developmental disturbances. Infants born to mothers with preeclampsia are more likely to exhibit difficult temperament traits, potential reflection of early manifestations of anxiety or mood disorders [21]. These children also are at increased risk of developing depression independent of gestational age or birth weight, and also for developing schizophrenia in adulthood, particularly those born preterm due to preeclampsia. The elevated production of tumor necrosis factor (TNF) observed in preeclampsia is thought to influence neuroglial processes and synaptogenesis, further contributing to the risk of psychiatric disorders such as schizophrenia [22,23,24].

Preeclampsia has been associated with an increased risk of allergic and severe atopic sensitizations, and a higher prevalence of asthma [25]. Additionally, term and preterm neonates born to mothers with preeclampsia exhibit a higher incidence of neonatal sepsis, which is thought to result from immune dysfunction and systemic inflammation related to this condition. Evidence suggests that preeclampsia may influence the development of the offspring’s immune system, either directly or indirectly through complications related to the “preeclamptic intrauterine environment” such as iatrogenic prematurity and increased rates of cesarean delivery [26,27].

Necrotizing enterocolitis (NEC) is a serious gastrointestinal disease predominantly affecting preterm and low birth weight infants. The pathophysiology of NEC is multifactorial, involving risk factors such as intestinal immaturity, hypoxia, formula feeding, and pathogenic bacterial colonization [28]. Premature neonates born to mothers with preeclampsia are at increased risk for developing NEC, which tends to manifest earlier and persist longer compared to those born to normotensive mothers [29]. Moreover, pediatric gastrointestinal conditions—such as hernias, functional colonic disorders, and esophageal complications, have been linked to severe forms of preeclampsia or eclampsia [30]. One explanation is the “predictive adaptive response” theory by Glickman et al. [31], which proposes that a compromised fetal environment leads to fetal programming through altered nutrient supply. This can cause permanent changes in gene expression via epigenetic mechanisms [30]. Such changes may increase susceptibility to diseases later in life, including gastrointestinal morbidities, if the postnatal environment does not match the fetal adaptations. Additionally, it is hypothesized that compromised fetal intestinal perfusion, aimed at preserving blood flow to other vital organs, leads to ischemic injury of the intestine, which may have long-term consequences for intestinal function [30].

The Barker Hypothesis (1993) proposed that maternal hypertension and placental ischemia during pregnancy may elevate the risk of cardiovascular diseases and stroke in the offspring. This theory has been substantiated through experimental and epidemiological data, that highlight the complex interplay between genetic predisposition and environmental influences in the pathogenesis of cardiovascular disease in children born to mothers with preeclampsia [32,33]. Renal dysfunction constitutes a critical aspect of this cardiovascular disease risk profile in offspring born to mothers with preeclampsia. Human nephrogenesis predominantly occurs during the third trimester—a period that frequently coincides with the onset of preeclampsia which affect unfavorably fetal kidney development [34]. Preeclampsia, often accompanied by intrauterine growth restriction and prematurity, is linked with reduced nephron number, decreased glomerular filtration rate, glomerular hypertrophy, and impaired renal vasodilation [35]. This nephron deficit is thought to result from placental dysfunction characteristic of preeclamptic pregnancies.

Furthermore, early-onset preeclampsia has been linked to alterations in the renin-angiotensin-aldosterone system (RAAS) in the offspring, which persist into adolescence [36]. Elevated aldosterone levels were observed in male adolescents born preterm to preeclamptic mothers, a finding that makes them predisposed to the development of hypertension later in life [37]. Prenatal exposure to preeclampsia was reported to be associated with a variety of alterations in the endocrine system of the offspring, influencing factors such as the development of obesity, adrenal gland function, salt sensitivity, androgen balance, and the timing and progression of puberty [38,39,40].

Evidence suggests that preterm neonates born to mothers with preeclampsia are at increased risk for developing severe respiratory distress syndrome (RDS) and bronchopulmonary dysplasia (BPD), and have a higher incidence of developing neonatal pneumonia [41,42,43]. Normal lung development depends on precise and coordinated signaling between the growing epithelium and its associated blood vessels, with lung angiogenesis playing a vital role in the proper formation of alveoli [44,45]. Because airway and alveolar growth occur in tandem with vascular development, the anti-angiogenic environment caused by preeclampsia during pregnancy may disrupt fetal lung maturation. This disruption could predispose extremely premature infants to more severe respiratory failure shortly after birth and contribute to abnormal development of the lung’s vascular and alveolar architecture over time. Furthermore, intrauterine exposure to preeclampsia was linked to an elevated lifetime risk for asthma and pulmonary hypertension [46,47]. Hypertensive disorders of pregnancy are established risk factors for the later development of chronic hypertension, which itself has been linked to respiratory diseases [48,49]. This suggests that common underlying mechanisms, such as vascular remodeling and endothelial dysfunction, may link these conditions [50]. Notably, when the onset of chronic hypertension following hypertensive disorders of pregnancy was taken into account, the increased risk of asthma and obstructive pulmonary disease was most pronounced among nurses who developed chronic hypertension after experiencing hypertensive disorders of pregnancy [51].

Preeclampsia was also associated with an increased risk of ocular morbidity, particularly in cases of severe preeclampsia or eclampsia [52]. Although the underlying mechanisms have not yet been fully elucidated, the placental hypoperfusion and fetal hypoxia induced by preeclampsia may upregulate the expression of hypoxia-inducible factor 1 (HIF-1), which was implicated in the endothelial dysfunction and visual system impairment [53]. Additionally, intrauterine stress resulting from preeclampsia, may trigger epigenetic modifications that increase the child’s long-term susceptibility to vascular diseases [54,55]. Moreover, iatrogenic preterm delivery—a frequent outcome in pregnancies complicated by preeclampsia—was identified as a risk factor for increased rate of ocular complications, including retinopathy of prematurity (ROP) [56]. Therefore, early diagnosis and appropriate management of preeclampsia, continuous monitoring of fetal health and prompt maternal treatment, are essential for the prevention of adverse outcomes and mitigating long-term risks for affected offspring.

### 2.2. Maternal Pre-Gestational Diabetes Mellitus and Gestational Diabetes Mellitus

The incidence of gestational diabetes mellitus (GDM) has been on the rise over time, currently affecting a substantial proportion of pregnancies worldwide [57,58,59]. GDM ranks ninth globally in terms of mortality rates. In 2021, approximately 537 million individuals were diagnosed with diabetes, of whom 239.7 million were individuals, unaware of having this condition. Additionally, a total of 6.7 million diabetes-related deaths were recorded globally. Regarding diabetes during pregnancy in particular, the global prevalence in 2021 reached 21.1 million cases. Among these, 80.3% were classified as GDM, 9.1% were other types of diabetes first identified during pregnancy, and 10.6% were pre-existing diabetes mellitus [60].

The prevalence of GDM varies by ethnicity and socioeconomic status. The lowest rates are observed among non-Hispanic White women (4.2%); Asian women are up to 11 times more likely to develop GDM compared to other ethnic groups [61,62,63,64,65]. Furthermore, seasonal variations in GDM incidence were reported, with higher rates observed during the summer months compared to the winter months [66]. The influence of ambient temperature on glucose tolerance may be related to the redistribution of blood between the arterial and venous systems due to changes in blood density. In regions with pronounced seasonal fluctuations, factors such as daylight duration, effects on photosensitive hormones (e.g., vitamin D, melatonin, serotonin), seasonal changes in diet, body weight, and physical activity, may also contribute to variations in GDM incidence [66]. The incidence of GDM increases in parallel with rising obesity rates, particularly in pregnant individuals with a body mass index (BMI) > 30 kg/m^2^ [66].

GDM is associated with an increased risk of pregnancy-related complications and has been implicated in adverse fetal and neonatal outcomes [66]. Pregnancies complicated with GDM are associated with increased morbidity and mortality of the fetus/neonate. Neonates born to GDM pregnancies exhibit 3-fold higher odds of developing congenital malformations such as fuel-mediated teratogenesis, congenital heart disease, sacral agenesis and left-sided microcolon. Furthermore, these fetuses are more susceptible to a deviant birth weight including either macrosomia (birthweight > 4.000 gr), or low birth weight (<2.500 gr). In cases of macrosomia there is increased risk of birth injuries and perinatal asphyxia during vaginal delivery, and due to that selective cesarean section is often performed [67]. Additional significant complications of GDM include premature birth, RDS, hypoglycemia immediately after birth, hypokalemia, hypomagnesemia, and hypocalcemia. There is also an increase in erythropoietin levels, leading to polycythemia, accompanied by enhanced destruction of red blood cells and subsequent hyperbilirubinemia. Infants of diabetic mothers are at increased risk for obesity during childhood and adolescence, and diabetes mellitus in the adulthood [67].

Recent studies highlight that maternal health status has a significant impact on the composition and functionality of the neonatal gut microbiome. Neonates born to mothers with GDM exhibit distinct alterations in their gut microbiota compared to those born to healthy mothers [67]. These alterations include reduced alpha and beta diversity, indicating a decreased variety and homogeneity of microbial populations, and changes in the relative abundance of specific bacterial taxa, potentially impacting critical functions of the microbiome. Early-life disturbances in the gut microbiota have been associated with an increased risk of developing inflammatory, allergic, and other metabolic disorders, and with alterations in the functionality of the immune system later in life [68]. These findings underscore the importance of maternal health during pregnancy in shaping the early microbial landscape and potentially influencing long-term health outcomes of the offspring [69,70].

Existing data showed that neonates of mothers with GDM, compared to those of non-GDM mothers, are predisposed to obesity in later life [69], and exhibit a significant reduction in gut microbial diversity, which may indicate a less resilient microbial environment [71]. In the long term, these neonates may be at increased risk for gastrointestinal and metabolic diseases [72,73]. Further studies have revealed that the oral microbiome of neonates born to mothers with GDM is enriched with specific bacterial species, suggesting potential effects of maternal metabolic status on the offspring’s microbial community. Moreover, GDM was associated not only with the structure and composition of the neonatal gut microbiome at distinct time points, but also with altered dynamic changes from birth through infancy. Thus, it is indicated that the impact of GDM on the neonatal microbiome may be immediate and progressive, influencing microbiota development during the critical early-life period. Therefore, altered colonization of the gut microbiome in infants born to mothers with GDM may affect their developmental trajectory [74]. These findings highlight the significant influence of GDM on early-life gut microbiome development and overall infant growth [59,74,75]. Monitoring these microbial shifts may contribute to a better understanding of the long-term implications of maternal health on microbial homeostasis and overall offspring health.

A systematic review and meta-analysis of 156 studies involving over 7.5 million pregnancies demonstrated that GDM is significantly associated with increased odds of several adverse perinatal outcomes, even after adjustment for confounding factors [76]. In women with GDM not treated with insulin, there was a higher risk of caesarean section, preterm birth, low 1 min Apgar score, macrosomia, and large-for-gestational-age (LGA) infants. Among insulin-treated cases, GDM was also linked to increased odds of neonatal respiratory distress syndrome, jaundice, LGA, and NICU admission. However, no consistent associations were observed with stillbirth, neonatal death, or low birth weight. These findings highlight the persistent burden of GDM-related complications despite treatment variations and underscore the importance of rigorous confounder adjustment in future studies.

A recent meta-analysis of 202 studies involving over 56 million mother–child pairs reported that maternal diabetes—gestational and pre-gestational—is significantly associated with an increased risk of neurodevelopmental disorders in the offspring, including autism spectrum disorder, Attention-Deficit/Hyperactivity Disorder (ADHD), intellectual disability, and various specific developmental, communication, motor, and learning disorders. These associations remained significant even after adjusting for multiple confounding variables. Pre-gestational diabetes had a stronger correlation with adverse neurodevelopmental outcomes compared to gestational diabetes [77]. These associations remained statistically significant even after adjustment for multiple confounding variables. In a subset of studies that performed such adjustments (*n* = 98; 49%), the pooled risk ratios (RRs) for offspring of diabetic mothers compared to unexposed children were as follows: any neurodevelopmental disorder (RR 1.28; 95% CI: 1.24–1.31), ASD (1.25; 1.20–1.31), ADHD (1.30; 1.24–1.37), intellectual disability (1.32; 1.18–1.47), specific developmental disorders (1.27; 1.17–1.37), communication disorders (1.20; 1.11–1.28), motor disorders (1.17; 1.10–1.26), and learning disorders (1.16; 1.06–1.26). Importantly, pre-gestational diabetes was more strongly associated with adverse neurodevelopmental outcomes (RR 1.39; 95% CI: 1.34–1.44) than gestational diabetes (RR 1.18; 95% CI: 1.14–1.23), with a statistically significant subgroup difference (*p*  <  0.0001). These findings underscore the critical importance of optimal maternal glycemic control during pregnancy and highlight the need for further high-quality longitudinal studies to clarify underlying causal pathways. Such evidence highlights the importance of maternal glycemic health in pregnancy and emphasize the need for further high-quality research to clarify causal relationships and mechanisms underlying these associations.

### 2.3. Congenital Heart Disease and Pregnancy (CHD)

In clinical practice, women with severe congenital heart disease (CHD) are increasingly able to successfully complete pregnancy and reach delivery with relatively low mortality rates. However, maternal and fetal morbidity and mortality risks still remain elevated [78]. The risk of neonatal death is approximately 3.5 times higher in neonates born to mothers with severe CHD compared to those born to mothers with milder forms of CHD [79]. Recently, Dhiman et al. [80] reported that the odds of FGR, preterm birth, low birth weight neonates, 5 min APGAR scores below 7, NICU admission rates, and congenital anomalies were all significantly higher in women with CHD compared to women without CHD. However, no neonatal mortality was observed in any of the groups.

Only a subset of congenital heart diseases is considered as a high-risk group during pregnancy, including Eisenmenger syndrome, cyanotic heart defects without prior surgical correction, stenotic lesions of the aorta, and Marfan syndrome [79]. Pregnant patients with cardiac disease are at increased risk of adverse fetal and neonatal outcome due to several contributing factors, such as maternal genetic predisposition, altered hemodynamic status, cardiac complications during gestation, and the use of cardiovascular medications that may affect fetal development [71,81,82,83]. Complications such as hypertensive disorders of pregnancy are frequently observed [84,85], while specific conditions like Fontan circulation are associated with an increased risk of postpartum hemorrhage [86]. Furthermore, patients with congenital heart disease exhibit a higher likelihood of transmitting the condition to their offspring raising the hypothesis that a woman with CHD may influence the fetal cardiac development of her offspring not only by transmitting her genes but also through a modified intrauterine environment [87,88,89]. Cases that involve fetal cardiac abnormalities are particularly associated with increased risks of preterm birth, low birth weight, and obstetric complications such as preeclampsia [87,90,91]. Crucial factors to these outcomes also include genetic predisposition, placental dysfunction, and an altered maternal-fetal environment [92].

### 2.4. Preterm Premature Rupture of Membranes (PPROM)

Preterm premature rupture of membranes (PPROM) occurs in approximately 2–3% of pregnancies and accounts for nearly one-third of preterm births, significantly contributing to perinatal morbidity and mortality. In pregnancies complicated by early PPROM—defined as rupture of fetal membranes prior to 37 weeks of gestation—neonatal mortality and morbidity are substantially increased [93]. The prognosis of such pregnancies is difficult to ascertain due to the influence of multiple factors, including the presence of clinically apparent intra-amniotic infection, gestational age at delivery, and the extent of oligohydramnios. Reported rates of chorioamnionitis and neonatal survival vary significantly in the literature. Chorioamnionitis is observed in 28–42% of pregnancies with PPROM, while neonatal survival rates range from 6.25% to 100%, depending on gestational age at birth [93].

In surviving neonates, the most severe complications include RDS, pulmonary hypoplasia, chronic lung disease, periventricular leukomalacia, intraventricular hemorrhage, and cerebral palsy. Data from a recent systematic review suggested that [94], PPROM at or near the threshold of viability is associated with significant obstetric and neonatal complications. One-third of pregnancies affected by this condition resulted in elective termination. In the remaining cases, the rate of live births was 65.9%, with a mean gestational age at delivery of 27.3 weeks and an average period from rupture to delivery at 39.4 days. The most common maternal complications included chorioamnionitis (reported in one-third of cases), endometritis, and postpartum hemorrhage. Neonatal mortality was reported in 23.9% of cases, while the most frequent neonatal complications were respiratory disorders (such as RDS, pulmonary hypoplasia, or pulmonary hypertension), ROP, sepsis, and intraventricular hemorrhage (IVH) [94]. Survival rates and survival without significant neurodevelopmental impairment at 2 years of age improved markedly with increasing gestational age, particularly from 22 to 28 weeks [94]. Given the significant perinatal morbidity and mortality associated with PPROM, particularly in early gestation, prevention, early diagnosis, and optimized management are essential. Prompt identification of risk factors, monitoring for signs of infection, and individualized perinatal care can improve neonatal outcomes and reduce long-term complications. Strengthening strategies to delay delivery when safely possible, and enhancing neonatal support play a critical role in improving survival and neurodevelopmental outcomes in affected infants.

### 2.5. Twin and Multiple Pregnancies

The incidence of multiple gestations has significantly increased over recent decades, primarily due to the widespread use of assisted reproduction technology and the increase of the average maternal age at pregnancy. In the United States, the rate of multiple gestations increased dramatically since 1980, reaching a peak in 2014, with a slight decline observed thereafter [95]. Between 1980 and 2009, the twin birth rate rose by 76%, with minor fluctuations, reaching 32.1 per 1000 live births in 2019 [96].

In contrast, the incidence of triplet and higher-order multiple pregnancies increased by more than 400% between 1980 and 1998. However, in 2019 this rate decreased by 55% from its peak, reaching a point of 0.87 per 1000 live births. This reduction is primarily attributed to the transfer of fewer embryos during in vitro fertilization procedures and to the increased use of fetal reduction techniques [96]. In the United States in 2013, perinatal and infant mortality rates were 5.3 per 1000 live births in singleton pregnancies, compared with 24.4, 61.1, and 137 per 1000 live births in twin, triplet, and quadruplet pregnancies, respectively [97]. A substantial proportion of multiple pregnancies are associated with preterm birth, low birth weight, fetal growth restriction (FGR), obstetric complications, and congenital anomalies. Neonates from twin pregnancies exhibit increased rates of perinatal morbidity and mortality and are at increased risk for a range of complications [98]. These include complications associated with prematurity, such as transient tachypnea of the newborn (TTN), RDS, bronchopulmonary dysplasia, patent ductus arteriosus, persistent pulmonary hypertension of the newborn, ROP, NEC, IVH, and long-term neurodevelopmental impairments, including cerebral palsy, learning disabilities, and other developmental disorders. Additional complications include congenital and chromosomal anomalies, as well as intrauterine growth restriction. Collectively, these conditions may necessitate admission of the newborn to the neonatal intensive care unit [98].

Although prenatal care and counseling for women with twin or higher-order multiple pregnancies are, in many respects, similar to those for singleton pregnancies, several important differences arise due to the increased risk of complications associated with multifetal gestations. Twin and higher-order multiple pregnancies are associated with increased risks for nearly all pregnancy-related complications, with the exceptions of fetal macrosomia [99]. Most complications, such as gestational hypertension and gestational diabetes, are managed similarly in singleton and multifetal pregnancies, as they typically affect both (or all) fetuses. However, in some cases, only one fetus may be affected, while the other(s) remain unaffected or less severely impacted. In such scenarios, the assessment of risks and benefits for any proposed intervention must be individualized according to the condition and prognosis of each fetus [99].

The most significant risk in twin pregnancies is preterm birth, which is the leading cause of increased perinatal mortality, neonatal morbidity, and long-term adverse outcomes. It is often difficult to distinguish morbidity specifically attributable to twinning from that due to prematurity. Additional contributors to poor outcomes include higher rates of intrauterine growth restriction and increased incidence of congenital anomalies [100]. The accurate determination of amnionicity and chorionicity is critical, as monochorionic twins (those sharing a single placenta) face unique and serious risks [100]. Shared placental circulation in monochorionic twins predisposes to several potentially life-threatening complications, such as twin-to-twin transfusion syndrome (TTTS), twin anemia–polycythemia sequence (TAPS), twin reversed arterial perfusion sequence (TRAP) and selective fetal growth restriction (sFGR). Monoamniotic twins who share a single amniotic sac, are at risk of umbilical cord entanglement and may, in rare cases, be conjoined. Due to the significantly increased risks in monochorionic twins of neurological morbidity and perinatal mortality compared with dichorionic twins, these pregnancies involving monochorionic or monoamniotic twins require intensified and specialized follow-up protocols, that differ from those employed in dichorionic twin pregnancies [100,101,102].

### 2.6. Pregnancies Complicated by Fetal Growth Restriction

FGR, also known as intrauterine growth restriction, is one of the most frequent and serious prenatal conditions, with significant implications for both pregnancy outcome and neonatal health. FGR is associated with a marked increase in perinatal mortality, and with neonatal complications such as preterm birth, respiratory distress, and intraventricular hemorrhage. Beyond its short-term impact on neonatal survival and health, FGR also carries long-term consequences [103,104,105,106]. Children born with FGR are at increased risk of neurodevelopmental disorders, including impairments in motor function, learning, and perception. Furthermore, these individuals have an increased likelihood of developing chronic conditions in adulthood, such as hypertension, diabetes mellitus, and cardiovascular disease [104,107]. Early diagnosis and close monitoring of pregnancies affected by FGR are vital for prompt interventions aimed at improving neonatal survival and long-term health outcomes. FGR constitutes a clinical entity linked to a wide spectrum of adverse prenatal and postnatal outcomes. Despite advances in modern medicine, delivery remains the only definitive treatment, although it does not fully resolve all associated complications. The development and implementation of standardized international protocols and guidelines for the early detection, comprehensive monitoring, and optimal management of pregnancies complicated by FGR are very important. Adoption of such protocols would facilitate evidence-based clinical practice and improve perinatal outcomes by minimizing complications and enhancing the prospects for the affected neonates [106].

### 2.7. Maternal Age (<18 and >36 Years)

A substantial body of research has evaluated obstetric and neonatal outcomes in relation to maternal age, with particular emphasis on women younger than 17 years or older than 35 years old. These studies highlight the risks and complications associated with adolescent and advanced maternal age, as these age groups exhibit increased susceptibility to a variety of obstetric and neonatal adverse outcomes [108]. Research indicates that adolescent mothers, particularly if under 17 years of age, are at elevated risk for anemia, eclampsia, fetal demise, preterm birth, and the delivery of low birth weight neonates. At the same time, they demonstrate higher rates of vaginal delivery and lower risks of preeclampsia and postpartum hemorrhage [109].

Conversely, advanced maternal age (≥35 years) has been associated with an increased risk of gestational diabetes, preeclampsia, placental insufficiency, higher rates of cesarean section, preterm birth, FGR, and elevated perinatal mortality [110]. In a recent epidemiological study by Kim et al. [111], the effect of maternal age on neonatal mortality was assessed, analyzing data from 2,161,908 pregnancies of the Korean Vital Statistics. The study concluded that, according to these national epidemiological data, a significantly increased risk of early neonatal mortality was observed only among mothers aged ≥40 years. In contrast, women aged 35–39 years—though categorized as being of advanced maternal age—did not exhibit an elevated risk for early neonatal mortality [111]. Similarly, Blomberg et al. [112], in a cohort study using data from the Swedish Medical Birth Register (1992–2010), reported that in a highly developed healthcare setting offering free prenatal care to all pregnant women, adolescent mothers exhibited a reduced risk of adverse obstetric and neonatal outcomes compared to the reference group. On the other hand, childbirth at an advanced maternal age was associated with a greater risk of complications for the mother and the neonate [112]. A pooled analysis of 22,188 mother–child pairs from five longitudinal cohorts across low- and middle-income countries (LMICs) revealed that both younger (≤19 years) and older (≥35 years) maternal age are associated with adverse perinatal and early life outcomes. Following adjustment for confounding variables, younger maternal age remained significantly associated with increased odds of low birthweight, preterm birth, stunting at 2 years, and lower educational attainment in the offspring. Although advanced maternal age was associated with an increased risk of preterm birth, it was concurrently linked to a lower likelihood of early childhood stunting and failure to complete secondary education. Notably, offspring of both maternal age extremes exhibited modest increases in fasting glucose concentrations in adulthood, suggesting potential long-term metabolic sequelae. These findings underscore the critical need to prevent adolescent pregnancies and to ensure long-term follow-up for children born to mothers at both extremes of reproductive age. This highlights the importance of age-tailored prenatal care and individualized obstetric management to improve maternal and neonatal outcomes across all age groups.

### 2.8. Maternal Substance Use During Pregnancy

The use of psychoactive and addictive substances—such as alcohol, tobacco, and certain prescription medications—during pregnancy constitutes a serious public health concern due to its significant short- and long-term effects. These effects encompass risks to neonatal health, impairment of maternal–infant bonding, and broader negative social implications [113,114,115]. Substance use during pregnancy has been linked to intrauterine fetal death, preterm birth, FGR, microcephaly, cardiac dysfunction, congenital anomalies, increased incidence of sudden infant death syndrome (SIDS), and the development of behavioral disorders in childhood [114,116]. Prevention and early intervention are essential for mitigating these adverse outcomes and safeguarding the well-being of the child and the family unit.

## 3. Congenital Viral Infections and Intrauterine Exposure to SARS-CoV-2

**Figure 2:**
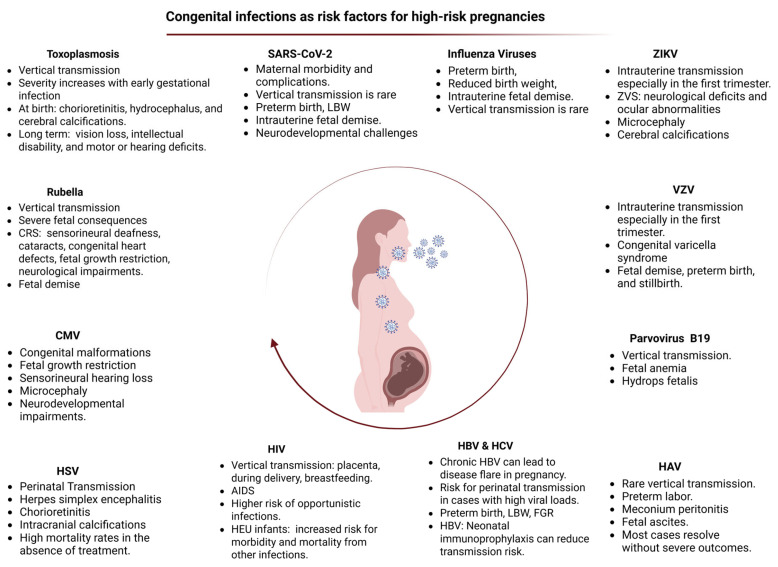
Visual summary of congenital viral infections and SARS-CoV-2 exposure during pregnancy. CMV: cytomegalovirus, CRS: congenital rubella syndrome, HSV: herpes simplex virus, HAV: hepatitis A virus, HBV: hepatitis B virus, HCV: hepatitis C virus, HIV: human immunodeficiency virus, AIDS: Acquired Immunodeficiency Syndrome, HEU: HIV exposed uninfected, ZVS: zika virus syndrome, LBW: low birth weight. Created in BioRender. L., A. https://BioRender.com/ymqw7ev (accessed on 5 June 2025).

Congenital viral infections, including intrauterine exposure to SARS-CoV-2, are increasingly recognized as significant risk factors contributing to high-risk pregnancies. These infections may lead to adverse perinatal outcomes such as preterm birth, fetal growth restriction, and potential long-term neurodevelopmental complications (Figure 2) [117,118,119].

### 3.1. Cytomegalovirus (CMV)

CMV is the most prevalent viral infection during pregnancy and is a major cause of adverse pregnancy outcomes, particularly congenital malformations and fetal growth restriction (FGR). Primary maternal infection in the first trimester poses the greatest risk, and vertical transmission leads to neurodevelopmental impairments such as sensorineural hearing loss, cognitive deficits, and microcephaly [118,120]. CMV crosses the placental barrier, resulting in placental inflammation, villous maldevelopment, and impaired trophoblast function, which collectively contribute to early-onset FGR. Although pre-existing maternal immunity reduces the risk of transmission but does not eliminate the possibility of reinfection or associated fetal complications [120,121]. CMV infection is also linked to delayed manifestations in infants, and its persistence in maternal fluids—including breastmilk—can lead to postnatal transmission, further highlighting the virus’s impact on maternal-fetal health [118].

### 3.2. Hepatitis Viruses

#### 3.2.1. Hepatitis A Virus (HAV)

HAV infection during pregnancy is uncommon, and vertical transmission is rare. However, isolated cases of fetal complications—such as meconium peritonitis, fetal ascites, and distal ileum perforation—were reported following maternal infection, necessitating surgical intervention in some instances [122,123]. While most maternal infections resolve without severe outcomes, acute HAV infection, particularly in the second or third trimester, were associated with adverse obstetric outcomes including preterm labor, placental abruption, and premature rupture of membranes, most likely mediated by maternal fever [123,124]. Neonatal impact is typically minimal, and breastfeeding is considered safe despite the presence of HAV RNA and antibodies in breast milk. Preventive strategies such as immunoglobulin administration and vaccination are effective in reducing transmission risk. Maternal anti-HAV IgG can cross the placenta and persist in the infant, influencing the timing and efficacy of vaccination, especially in high-endemic regions [118].

#### 3.2.2. Hepatitis B Virus (HBV)

HBV infection in pregnancy may lead to adverse maternal outcomes, particularly in women with chronic HBV who may experience disease flare up due to pregnancy-related immune modulation, marked by elevated HBV DNA levels and liver enzymes [125]. While acute or chronic HBV infection alone does not significantly increase fetal complications, maternal liver cirrhosis or failure can increase maternal morbidity and mortality. The primary fetal risk is perinatal transmission, especially in mothers with high viral loads (>200,000 IU/mL) [126]. Antiviral therapy during the third trimester, along with neonatal immunoprophylaxis using HBV vaccine and hepatitis B immune globulin (HBIG), significantly reduces the risk of vertical transmission [118,127].

#### 3.2.3. Hepatitis C Virus (HCV)

HCV infection during pregnancy is associated with an increased risk of adverse perinatal outcomes, including preterm delivery, FGR, congenital anomalies, cholestasis, and perinatal mortality [128,129]. While chronic HCV infection alone does not typically worsen pregnancy outcomes, maternal immune alterations during pregnancy may augment viral replication. Vertical transmission occurs in approximately 5% of cases and may be exacerbated by co-infection with HIV-1 [130]. Although breastfeeding and delivery mode do not significantly affect transmission risk, infection of placental or peripheral blood mononuclear cells may facilitate maternal-fetal transmission [129]. Maternal HCV infection may also impact placental morphology and immune cell activation, further contributing to pregnancy complications. Ribavirin, a key antiviral agent, is contraindicated in pregnancy due to its teratogenicity [130].

### 3.3. Human Immunodeficiency Virus (HIV)

Despite effective antiretroviral therapies, HIV remains a significant risk for maternal and neonatal health, and approximately 38 million people are infected globally, half of whom are women. Vertical transmission of HIV through the placenta, during delivery, or through maternal milk, leads to congenital HIV infection, the primary cause of neonatal HIV-related morbidity and mortality. Neonates born to HIV-positive mothers are at a increased risk for transmission, especially in the absence of antiretroviral therapy, with a 25% transmission rate leading to severe neonatal outcomes including AIDS and increased susceptibility to cardiovascular and opportunistic infections [119,131]. Furthermore, HIV-exposed uninfected infants (HEU) have a significantly higher risk of morbidity and mortality from infections such as diarrhea and respiratory illnesses. These risks are influenced by factors such as maternal disease severity, poor placental antibody transfer, and administration of antiretroviral drugs [119,130].

### 3.4. Toxoplasmosis

Toxoplasmosis is a globally prevalent parasitic disease caused by *Toxoplasma gondii*, an obligate intracellular protozoan. It is estimated that approximately one-third of the world’s population has been exposed to the parasite [132]. Three major genotypes of *T. gondii* (types I, II, and III) have been identified, with genotype II being predominant in Europe (95%) and accounting for 43.9% of infections in North America. In contrast, strains in South America demonstrate greater genetic diversity and are associated with markedly increased virulence [133,134,135,136]. More recently, a fourth clonal lineage (type 12) has been detected in wildlife populations in North America [137]. Congenital toxoplasmosis results from primary maternal infection acquired during pregnancy. During parasitemia, *T. gondii* can cross the placenta and invade the fetal circulation. The risk of vertical transmission increases with advancing gestational age—estimated at 2.2% at 6 weeks, 23% at 18 weeks, and 56% at 30 weeks of gestation [138,139]. However, the severity of fetal disease is inversely correlated with gestational age at the time of infection. First-trimester infections are associated with the highest risk of adverse outcomes, including miscarriage (~3% of cases), intrauterine fetal demise, and severe congenital abnormalities such as microcephaly, hydrocephalus, intracranial calcifications, hepatosplenomegaly, and sensorineural visual or auditory deficits [138,140]. Although the risk of fetal infection peaks in the third trimester, neonatal presentations are more often mild or asymptomatic [141,142]. The global seroprevalence of *Toxoplasma gondii* among pregnant women has been estimated at 36.6% (95% CI: 33.7–39.6), based on meta-analytic data [143]. The highest regional prevalence was observed in South America (52.8%; 95% CI: 46.6–59.0), followed by Africa (46.8%; 95% CI: 39.5–54.3), whereas the lowest rates were reported in North America (19.7%; 95% CI: 8.4–39.6) and Europe (24.6%; 95% CI: 17.8–32.9) [143]. The incidence of acute *T. gondii* infection among seronegative pregnant women varies geographically, with rates reported at 0.8/1000 in Austria, 0.5/1000 in Sweden, 2.1/1000 in France, and 0.2/1000 in the United States [144,145,146]. Globally, the incidence of congenital toxoplasmosis is estimated at 1.5 per 1000 live births, with higher prevalence noted in South America, parts of the Middle East, and low-income regions [147]. The precise mechanisms underlying vertical transmission remain incompletely understood. The placenta exhibits cell-type-specific resistance, with syncytiotrophoblasts offering greater protection compared to cytotrophoblasts and extravillous trophoblasts. Placental immune responses, including the induction of cytokines and chemokines, are believed to play a pivotal role in modulating susceptibility and fetal invasion [148]. Timely identification and dating of maternal infection are essential for risk stratification, prenatal management, and counseling, particularly given the significant variation in fetal outcomes based on the gestational age at transmission [138,140].

### 3.5. Rubella

Vertical transmission of rubella virus (RV) during pregnancy can result in severe fetal consequences, particularly when maternal infection occurs in the first trimester. The virus crosses the placenta, replicates in placental tissues, and causes placentitis, leading to fetal infection and widespread cellular damage [118]. Early gestational infections (especially within the first 8 weeks) carry a high risk of congenital rubella syndrome (CRS), characterized by sensorineural deafness, cataracts, congenital heart defects, fetal growth restriction, and neurological impairments [149]. Miscarriage occurs in up to 20% of early infections, and approximately 75% of fetuses are affected. While the risk and severity of outcomes decline with advancing gestation, late-onset sequelae such as cardiac defects, developmental delay, and neuropsychiatric disorders may still occur [118].

### 3.6. Herpes Simplex Virus

Herpes simplex virus (HSV) infection during pregnancy, though often asymptomatic or mild in the mother, poses significant risks to the neonate, particularly when maternal infection occurs near delivery. Vertical transmission typically occurs through contact with genital lesions during birth rather than transplacental spread [150]. Neonatal HSV infection can lead to severe complications such as herpes simplex encephalitis, chorioretinitis, and intracranial calcifications, with mortality rates reaching 50–80% in the absence of treatment. Alterations in maternal immunity during pregnancy may also increase susceptibility to HSV infection [118,119].

### 3.7. Parvovirus

Parvovirus B19 infection during pregnancy poses a significant risk for vertical transmission, particularly in the first trimester, when fetal susceptibility is highest. Transmission rates during acute maternal infection range from 17% to 33%, and fetal consequences are most severe when infection occurs before 20 weeks of gestation [151]. The virus targets fetal erythroblasts, leading to profound anemia, hydrops fetalis, and potentially fetal death in 4% to 10% of cases. Hydrops results from cardiac failure secondary to severe anemia [118,119]. Parvovirus B19 represents the sole known treatable cause of non-immune hydrops fetalis. Management typically involves administering intrauterine blood transfusions to treat the severe fetal anemia. This intervention provides a critical window for the developing fetal immune system or maternal antibodies to mount a response against the infection [152]. Newborns affected by parvovirus B19 often test positive for viral DNA at birth and subsequently generate their own antibody response to the virus [118].

### 3.8. Varicella Zoster Virus (VZV)

VZV can be vertically transmitted during pregnancy, delivery, or the postpartum period, although the precise mechanism of in utero transmission remains unclear. Intrauterine infection is supported by the detection of VZV DNA and replication markers in placental and fetal tissues, as well as the presence of virus-specific antibodies in symptomatic neonates at birth [153]. The virus is believed to disseminate via infected T cells reaching the basal decidua and spreading into placental circulation [154]. VZV is teratogenic, with first-trimester infections posing the highest risk for congenital varicella syndrome (CVS), while infections later in pregnancy are rarely associated with fetal anomalies. Zoster during pregnancy does not appear to cause CVS, likely due to maternal immunity. However, VZV infection during pregnancy can lead to severe maternal complications, including respiratory illness, miscarriage, preterm birth, and stillbirth [118,154].

### 3.9. Zika Virus (ZIKV)

Zika virus is epidemic in the Americas, primarily linked to strains of the Asian lineage, and is associated with a spectrum of fetal anomalies termed congenital Zika virus syndrome (ZVS), including microcephaly, cerebral calcifications, neurological deficits, and ocular abnormalities [155]. Transmission occurs mainly via Aedes aegypti mosquitoes, though sexual transmission was also documented. Evidence of transplacental transmission is supported by the detection of viral RNA in fetal tissues, placenta, and amniotic fluid [118]. The risk of congenital malformations is highest following first-trimester infections and declines in later gestation. Disparities in rates of fetal abnormalities may reflect contributing factors such as maternal undernutrition, urban crowding, and limited preventive awareness. ZIKV targets various placental cell types and its ability to replicate in trophoblasts and Hofbauer cells may facilitate fetal infection [156]. Comparative studies suggest that the American strains are more likely to support placental invasion and vertical transmission than African strains, potentially due to differences in immune response induction, cell tropism, and viral replication kinetics [118,119,130].

### 3.10. Influenza Viruses

Influenza viruses pose a significant global health threat due to their capacity for rapid antigenic evolution and pandemic potential, necessitating continuous vaccine updates. Pregnant women, particularly in the third trimester, are disproportionately susceptible to severe influenza outcomes, including increased mortality and adverse fetal effects, such as intrauterine fetal demise, reduced birth weight, and preterm delivery, largely driven by immunological and physiological alterations during pregnancy. These effects are likely mediated indirectly, as vertical transmission of the virus is rarely observed [119,130]. While adaptive immune responses, including vaccination-induced seroconversion, appear relatively preserved, innate responses—such as interferon signaling and cytokine regulation—are often impaired. Animal studies reinforce these findings, demonstrating heightened mortality and inflammation in pregnant mice [157]. Hormonal influences, particularly estradiol, estriol, and progesterone, appear protective, though the role of other pregnancy-altered hormones remains unclear. Despite substantial research, critical gaps persist in the understanding of the molecular mechanisms underlying altered immunity in pregnancy, underscoring the need for targeted investigation to inform improved therapeutic and preventive strategies for this vulnerable population [130].

### 3.11. Intrauterine Exposure to SARS-CoV-2

The COVID-19 pandemic significantly impacted global public health, with particular concern regarding its effects on pregnancy, both in healthcare professionals and pregnant individuals. Research conducted during the pandemic revealed that pregnant individuals infected with SARS-CoV-2 face an elevated risk of serious complications [158]. Pregnant individuals with COVID-19 are at increased risk of severe illness and are more likely to require hospitalization in an intensive care unit (ICU), mechanical ventilation, or intubation. SARS-CoV-2 infection was associated with raised fetal risks including higher rates of preterm birth, low birth weight, and intrauterine fetal demise. Although mother-to-fetus transmission is considered rare, the potential of vertical transmission remains an area of active investigation. There is also a risk of developing complications due to maternal comorbidities with pregnancies complicated by pre-existing conditions (e.g., diabetes, hypertension), being at risk for worse outcomes following SARS-CoV-2 infection [158].

Although most pregnant women infected with SARS-CoV-2 exhibit mild to moderate symptoms, they are at increased risk for severe disease compared to the general population of reproductive-aged women [158]. Available data suggest that vertical transmission of SARS-CoV-2 is possible, but at very low rates [159,160]. Documented cases of placental transmission are rare and based on limited but compelling evidence. Estimated transmission rates range from 1.6% to 6.3%, although the precise rate has not yet been fully evaluated [161,162]. Neonatal infection with SARS-CoV-2 is uncommon, and most affected neonates exhibit minimal clinical symptoms, if any [163]. Nonetheless, concerns persist regarding the potential impact of maternal infection placental function and fetal development. Several studies reported increased rates of preterm delivery, low birth weight, and NICU admission following maternal SARS-CoV-2 infection during pregnancy [164,165]. It remains unclear whether these NICU admissions are attributable to placental pathology, or to increased rates of preterm birth in this population.

Histopathological examination of placentas from pregnancies affected by COVID-19 demonstrated abnormalities in maternal and fetal vascular circulation [166,167,168,169]. These findings occur in the absence of inflammatory infiltrates or confirmed placental infection, suggesting that placental lesions may reflect a maternal hypercoagulable or hyperinflammatory state triggered by COVID-19. Reports of “COVID-19 placentitis”—a severe, diffuse or focal placental lesion resulting from direct viral infection—have emerged, characterized by massive perivillous fibrin deposition and chronic intervillositis [170,171,172,173,174].

Intrauterine inflammation may result in adverse pregnancy outcomes, including preterm birth and intrauterine fetal demise [175]. The cytokine cascade associated with severe maternal COVID-19 may adversely affect pregnancy outcomes and contribute to postpartum complications. Pregnant individuals infected with SARS-CoV-2 exhibit a systemic inflammatory response, with elevated circulating levels of cytokines such as IL-8, IL-10, and IL-15 [176]. Even asymptomatic individuals often present with lymphopenia, including reduced T-cell subsets critical for maintaining maternal–fetal immune tolerance, potentially compromising fetal immunoprotection. Additionally, immunologic alterations were detected in placental tissues from infected individuals, even in the absence of detectable viral RNA [176]. Elevated levels of pro-inflammatory cytokines such as IL-6, IL-8, and interferon-gamma-induced protein 10 (IP-10) were also identified in blood samples of neonates born to SARS-CoV-2-infected mothers, suggesting a state of perinatal systemic inflammation [176,177]. There is increasing evidence that maternal SARS-CoV-2 infection not only triggers a maternal cytokine response but may also induce fetal inflammatory responses, even without direct placental infection [176]. Preliminary findings from several studies suggest an association between prenatal exposure to SARS-CoV-2 and poor motor and socio-emotional developmental outcomes in infancy [178,179]. One analysis of 222 infants born to mothers with SARS-CoV-2 infection during pregnancy, compared to 7550 controls born during the same period, reported significantly higher rates of neurodevelopmental disorders in the exposed group. This association was particularly evident among infants exposed during the third trimester of intrauterine life, suggesting increased fetal vulnerability during this critical developmental stage [180]. Most diagnoses were related to motor, speech, and language delays. Although an increased rate of preterm birth was observed in SARS-CoV-2-positive mothers, statistical adjustment for confounding factors, including prematurity, confirmed a significant independent association between in utero exposure to SARS-CoV-2 and any neurodevelopmental complication.

Additionally, increased rates of respiratory distress syndrome was reported in term neonates exposed to, but not infected by, SARS-CoV-2 [181]. Elevated maternal inflammatory cytokine levels during gestation may adversely affect fetal brain and lung development, disrupting key elements of normal organ maturation [182,183]. In a cohort study of 896 children, repeated neurodevelopmental assessments revealed no substantial association between prenatal SARS-CoV-2 exposure and neurodevelopment during the first two years of life [184]. However, a recent meta-analysis [185] reviewing outcomes of neonates with in utero exposure to SARS-CoV-2, highlighted the limited and conflicting nature of current evidence regarding the association between maternal SARS-CoV-2 infection and short- or long-term neonatal morbidity. The strongest evidence supports a relationship between maternal infection and increased risk of respiratory complications linked to preterm birth, while associations with neurological or gastrointestinal morbidity remain unsubstantiated [185]. The authors of the meta-analysis emphasized the urgent need for longitudinal studies to clarify the long-term effects of maternal SARS-CoV-2 infection on infant neurodevelopment [185]. Additionally, there is a need for standardized, validated neurodevelopmental assessment tools to evaluate long-term outcomes across diverse populations.

Given the above findings, long-term follow-up of all neonates born to mothers infected with SARS-CoV-2 during pregnancy is essential, even in the absence of symptoms at birth. Such monitoring is critical for early detection of potential developmental or neurological impairments, positioning these infants as a high-risk population in the COVID-19 era. Systematic surveillance using standardized neurodevelopmental evaluations can facilitate timely diagnosis and intervention, potentially mitigating adverse long-term outcomes.

## 4. Additional Risk Factors Defining a High-Risk Pregnancy and Their Neonatal Impact

The management of HRPs necessitates a coordinated, multidisciplinary approach to reduce maternal and fetal complications and to optimize perinatal outcomes. These pregnancies are inherently associated with an increased likelihood of adverse events, including preterm birth, FGR, congenital anomalies, and the need for admission to a NICU. Furthermore, affected neonates are at elevated risk for long-term neurodevelopmental impairments and metabolic disorders. Beyond well-established etiologies—such as preeclampsia, GDM, and intrauterine infections—a range of additional maternal, obstetric, genetic, environmental, and social factors may independently define a pregnancy as high risk. These risk factors, individually or in combination, can significantly influence neonatal morbidity and mortality. The most clinically relevant of these factors are summarized in the Table 1.

## 5. Discussion

The monitoring and management of high-risk pregnancies require a multidisciplinary approach to minimize associated complications and achieve optimal outcomes for the mother and fetus. Multiple maternal conditions have been identified as significant contributors to adverse neonatal outcomes. Preeclampsia, affecting 3–8% of pregnancies, is strongly associated with preterm birth, FGR, and NICU admission, with neonates at elevated risk for neurodevelopmental disorders, schizophrenia, sepsis, and renal abnormalities [21,41,42,43]. Similarly, GDM increases the risk of congenital anomalies, macrosomia, neonatal hypoglycemia, respiratory distress syndrome (RDS), and future obesity, with emerging evidence pointing to altered gut microbiota and inflammatory programming in the offspring. Studies assessing prevention and management strategies for GDM highlight its long-term impact on offspring health. Maternal hyperglycemia has been linked to persistent metabolic alterations in children, influenced by epigenetic changes and disruptions in gut microbiota composition. Offspring exposed to GDM are at higher risk for developing obesity, impaired glucose metabolism, and cardiovascular conditions later in life [230]. Early detection through prenatal screening, along with timely lifestyle interventions and continued monitoring postnatally, are essential to mitigate these risks. These insights underscore the need for an integrated maternal care approach aimed at improving health trajectories across generations. Severe maternal congenital heart disease elevates the risk of neonatal mortality (3.5-fold), low birth weight, preterm birth, and structural anomalies, primarily due to compromised maternal hemodynamics and medication exposure [79,87]. PRM accounts for up to one-third of preterm deliveries and is linked to RDS, sepsis, IVH, and cerebral palsy, with chorioamnionitis significantly contributing to long-term neurodevelopmental deficits [94]. Multiple pregnancies are associated with markedly increased perinatal mortality and morbidity, including higher rates of RDS, necrotizing enterocolitis, IVH, and retinopathy of prematurity. Monochorionic twins, in particular, are vulnerable to complications such as TTTS [98,99]. Fetal growth restriction is a key predictor of perinatal death, RDS, and adult-onset cardiometabolic disease, reflecting the lasting impact of early-life undernutrition and hypoxia [103,104,105,106].Extremes of maternal age carry distinct risks: teenage pregnancies are associated with FGR and neonatal death, whereas advanced maternal age increases the incidence of GDM, preeclampsia, cesarean delivery, and early neonatal mortality [108]. Maternal substance use has been linked to FGR, congenital anomalies, neurobehavioral disorders, and SIDS, requiring intensive care and long-term follow-up [114,116]. Lastly, intrauterine infection, including SARS-CoV-2 exposure, has been associated with prematurity, NICU admission, and altered neurodevelopment, even in the absence of vertical transmission, highlighting the significance of the fetal inflammatory environment [117,118,119].

Numerous additional risk factors contribute to the classification of a pregnancy as high-risk. These include maternal obesity or severe weight loss, both of which may lead to metabolic disorders and gestational complications [58,59], renal diseases which can adversely affect fetal development and pregnancy progression [9], and a history or presence of mental health conditions, such as depression and anxiety disorders [231]. Immunological disorders, such as antiphospholipid syndrome, are also significant, as they are associated with increased risk of thrombosis and fetal demise [232]. Excessive caffeine intake and poor maternal nutrition can negatively impact maternal and fetal health. Environmental and occupational hazards, including exposure to toxic substances or engagement in strenuous manual labor, further increase the risk for adverse outcomes. Genetic anomalies or hereditary disorders, whether from the maternal or paternal site, also play a critical role [233]. Grand multiparity, defined as more than five previous deliveries, is another important consideration. A history of pregnancy-related complications, including spontaneous or induced abortions, preterm births, and intrauterine fetal demise, increases the likelihood of adverse outcomes in subsequent pregnancies [234,235]. Surgical complications arising during gestation, as well as anomalies and infections of the urogenital tract, present additional concerns. Maternal febrile illnesses and infections such as toxoplasmosis, rubella, syphilis, cytomegalovirus, hepatitis B or C, and HIV have established associations with adverse fetal outcomes [118].

Previous cesarean sections or other uterine interventions are factors which may also complicate a subsequent pregnancy [235]. Other medical conditions, such as anemia, Rhesus sensitization, ABO incompatibility, and endocrine disorders, further exacerbate the risks [16]. Autoimmune diseases and intrahepatic cholestasis of pregnancy are also notable contributors. Gestational bleeding, particularly in the context of placenta previa or placental abruption, constitutes a medical emergency with severe implications for fetal viability, while uterine or vaginal malformations may also interfere with normal gestation and delivery [236]. Low socioeconomic status and inadequate prenatal care, defined as fewer than three antenatal visits, are recognized as social determinants that significantly impact perinatal outcomes [237]. History of obstetric interventions or birth trauma further contributes to the classification of a pregnancy as high-risk.

Despite growing recognition of the impact of pregnancy-specific conditions—such as preeclampsia, gestational diabetes, and peripartum cardiomyopathy—on both maternal and neonatal outcomes, significant gaps persist in the evidence base guiding clinical care. Data limitations, including inconsistent outcome reporting and underrepresentation of pregnant individuals in clinical trials, continue to hinder progress in understanding disease mechanisms, evaluating interventions, and improving perinatal care. One critical challenge is the exclusion of pregnant women from many therapeutic trials, which restricts the development of safe and effective pharmacologic treatments for conditions that can have lifelong consequences for both mother and child. The lack of approved therapies reflects broader barriers in the drug development pipeline, from inadequate preclinical models to regulatory and ethical constraints in trial design [238,239]. Addressing these gaps will require increased investment in preclinical research, particularly into placental biology and early pregnancy physiology, alongside improved use of pharmacokinetic modeling and real-world data. Moreover, further outcomes-based research is needed to better characterize how maternal comorbidities—such as cardiovascular disease, hemorrhage, and hypertensive disorders—interact with pregnancy and how targeted interventions might alter maternal and neonatal trajectories. This includes evaluating not only biomedical treatments but also social, demographic, and health system factors contributing to disparities, particularly among racially and socioeconomically marginalized populations. To advance the field, a coordinated, cross-disciplinary approach is essential. This includes forming networks that integrate researchers, clinicians, policy makers, and patients to drive innovation in maternal health. Enhancing training and regulatory support for pregnancy-focused research, expanding the availability of high-quality longitudinal data, and advocating for prioritization of maternal health by funding bodies and academic institutions will be key to filling current knowledge gaps and improving outcomes across generations [240,241].

## 6. Conclusions

A substantial proportion of high-risk pregnancies is associated with increased rates of preterm birth, low birth weight, intrauterine growth restriction, and congenital anomalies. These complications are linked to significantly increased rates of perinatal mortality and are associated with neurodevelopmental disorders and an increased risk of chronic disease in adult life. Evidence from clinical research focusing on short- and long-term outcomes of high-risk pregnancies demonstrates that preterm birth is the most frequent and severe complication, exerting a profound effect on the child’s health and development. The mortality, morbidity, and long-term prognosis of preterm neonates are influenced not only by gestational age at birth, birth weight, and the level of neonatal care provided, but also by medical conditions that precipitated the preterm delivery [242]. Early identification of women at increased risk for pregnancy complications is critically important. In some cases, prompt recognition allows the implementation of therapeutic interventions aimed at mitigating adverse outcomes. In others, closer surveillance or inclusion in clinical research protocols may be warranted to enhance understanding and improve management strategies for these complex conditions. Ensuring the safety of pregnancy and motherhood process, requires a holistic approach that integrates high-quality medical care with attention to the broader socioeconomic conditions that critically influence the health outcomes of both the mother and the offspring.

Key components of an effective maternal health strategy include comprehensive family planning, widespread prenatal screening, systematic pregnancy monitoring, and prompt identification of high-risk pregnancies. High-risk pregnancies should be referred to specialized fetal-maternal medicine centers to ensure appropriate monitoring and effective prevention of complications. These measures can significantly contribute to the reduction of maternal and perinatal mortality and morbidity, ultimately promoting health and well-being of the dyad mother and child.

## Figures and Tables

**Table 1:** Maternal diseases and risk factors with associated pregnancy complications and neonatal outcomes.

Maternal Disease/Risk Factor		Pregnancy Complications and Neonatal Outcomes		References
**Metabolic disease**	Obesity/Severe weight loss	Pregnancy	Increases risk of gestational diabetes, preeclampsia, dystocia, and cesarean delivery.	[186,187,188]
		Offspring	Macrosomia or fetal growth restriction (FGR), shoulder dystocia, neonatal hypoglycemia, increased neonatal intensive care unit (NICU) admissions.	
**Renal disease**	Chronic kidney disease, acute kidney injury	Pregnancy	Linked with hypertension, preeclampsia.	[189,190,191,192,193]
		Offspring	Preterm birth, low birth weight, FGR, increased perinatal mortality.	
**Mental Health**	History of depression/anxiety	Pregnancy	Elevated risk of postpartum depression.	[194,195,196]
		Offspring	Preterm birth, low Apgar score impaired maternal-infant bonding, and increases long-term cognitive/behavioral issues	
**Immunologic Disorders**	Antiphospholipid syndrome, autoimmune rheumatic diseases, systemic lupus erythematosus (SLE), inflammatory bowel disorders	Pregnancy	High risk for miscarriage, stillbirth, preeclampsia, and maternal thrombosis.	[197,198,199,200,201,202,203]
		Offspring	FGR, preterm birth, increased neonatal susceptibility to infections, suboptimal vaccine responses, potential organ toxicity, congenital malformations neonatal lupus, congenital heart block, and adverse long-term outcomes such as metabolic and cardiovascular diseases as well as neurodevelopmental impairments and autism spectrum disorder.	
**Nutritional status**	Inadequate maternal nutrition, maternal under- and over-nutrition	Pregnancy	Placental insufficiency, and metabolic dysregulation preeclampsia, anemia, increased maternal mortality	[204,205,206,207,208]
		Offspring	FGR, preterm birth, small for gestational age (SGA), low birth weight, neural tube defects, increased neonatal susceptibility to infections, neonatal hypothermia, elevated risk of neonatal death, and long-term health complications, including obesity, type 2 diabetes mellitus, hypertension, and cognitive impairments.	
**Environmental/Occupational**	Exposure to chemicals, heavy labor	Pregnancy	Risk of embryotoxicity, miscarriage, preterm delivery, and impaired placental oxygenation.	[209,210,211]
		Offspring	Congenital anomalies, prematurity and low birth weight	
**Genetic**	Hereditary diseases/family history	Pregnancy	Increased risk for, miscarriage and perinatal loss and preterm birth, risk of maternal HELLP syndrome or acute fatty liver of pregnancy	[212,213]
		Offspring	Preterm birth, congenital anomalies and inherited disorders in the fetus.	
**Obstetric History**	Uterine anomalies, cervical insufficiency, previous preterm labor, miscarriages, stillbirth, uterine surgery	Pregnancy	Associated with cervical insufficiency, cord prolapse, uterine rupture, placenta accreta, and intrapartum hemorrhage and placenta previa, miscarriage, preterm labor, abnormal fetal presentation. Raises the likelihood of complications in subsequent deliveries (e.g., dystocia).	[214,215,216,217]
		Offspring	Birth injuries, prematurity, perinatal asphyxia, congenital abnormality.	
**Hematologic**	Anemia, Thrombophilia, Rh isoimmunization,	Pregnancy	Preeclampsia, placental abruption, coagulation abnormalities, increased risk of thrombosis, preterm delivery.	[218,219,220]
		Offspring	Hemolytic disease of the newborn, neonatal anemia, FGR, fetal loss, fetal anemia, edema, thrombosis-related neonatal complications, neonatal jaundice, increased NICU admissions	
**Liver/Biliary**	Intrahepatic cholestasis of pregnancy	Pregnancy	Increased risk of stillbirth, preterm labor, post-partum hemorrhage, and emergency caesarean section risk	[221,222,223]
		Offspring	Stillbirth, meconium-stained amniotic fluid, respiratory distress, NICU admission, perinatal death	
**Thyroid dysfunction**	hyperthyroidism, hypothyroidism and thyroid autoimmune disorders	Pregnancy	Pregnancy induced hypertension thyroid crisis, preeclampsia, preterm labor, miscarriage, postpartum hemorrhage	[224,225,226]
		Offspring	Congenital anomalies (cardiac, neural tube), macrosomia, preterm birth, hypoglycemia, neurodevelopmental delays	
**Social Factors**	Low socioeconomic status Inadequate prenatal follow-up (<3 visits)	Pregnancy	Linked to higher maternal morbidity and mortality, inadequate prenatal care, malnutrition.	[227,228,229]
		Offspring	Increases the risk of undetected complications, low birth weight, prematurity, neonatal infection, hypothermia, increased risk of perinatal and neonatal mortality	

## Data Availability

Data are contained within the article.
